# CDO1 Promoter Methylation is a Biomarker for Outcome Prediction of Anthracycline Treated, Estrogen Receptor-Positive, Lymph Node-Positive Breast Cancer Patients

**DOI:** 10.1186/1471-2407-10-247

**Published:** 2010-06-01

**Authors:** Dimo Dietrich, Manuel Krispin, Jörn Dietrich, Anne Fassbender, Jörn Lewin, Nadia Harbeck, Manfred Schmitt, Serenella Eppenberger-Castori, Vincent Vuaroqueaux, Frédérique Spyratos, John A Foekens, Ralf Lesche, John WM Martens

**Affiliations:** 1 Epigenomics AG, Berlin, Germany; 2 Breast Center, University of Cologne, Germany; 3 Technical University Munich, Germany; 4 Stiftung Tumorbank, Basel, Switzerland; 5 Institute of Pathology, University Basel, Switzerland; 6 Centre René Huguenin, St. Cloud, France; 7 Erasmus MC, Josephine Nefkens Institute and Cancer Genomics Centre, Rotterdam, the Netherlands

## Abstract

**Background:**

Various biomarkers for prediction of distant metastasis in lymph-node negative breast cancer have been described; however, predictive biomarkers for patients with lymph-node positive (LNP) disease in the context of distinct systemic therapies are still very much needed. DNA methylation is aberrant in breast cancer and is likely to play a major role in disease progression. In this study, the DNA methylation status of 202 candidate loci was screened to identify those loci that may predict outcome in LNP/estrogen receptor-positive (ER+) breast cancer patients with adjuvant anthracycline-based chemotherapy.

**Methods:**

Quantitative bisulfite sequencing was used to analyze DNA methylation biomarker candidates in a retrospective cohort of 162 LNP/ER+ breast cancer patients, who received adjuvant anthracycline-based chemotherapy. First, twelve breast cancer specimens were analyzed for all 202 candidate loci to exclude genes that showed no differential methylation. To identify genes that predict distant metastasis, the remaining loci were analyzed in 84 selected cases, including the 12 initial ones. Significant loci were analyzed in the remaining 78 independent cases. Metastasis-free survival analysis was conducted by using Cox regression, time-dependent ROC analysis, and the Kaplan-Meier method. Pairwise multivariate regression analysis was performed by linear Cox Proportional Hazard models, testing the association between methylation scores and clinical parameters with respect to metastasis-free survival.

**Results:**

Of the 202 loci analysed, 37 showed some indication of differential DNA methylation among the initial 12 patient samples tested. Of those, 6 loci were associated with outcome in the initial cohort (n = 84, log rank test, p < 0.05).

Promoter DNA methylation of cysteine dioxygenase 1 (CDO1) was confirmed in univariate and in pairwise multivariate analysis adjusting for age at surgery, pathological T stage, progesterone receptor status, grade, and endocrine therapy as a strong and independent biomarker for outcome prediction in the independent validation set (log rank test p-value = 0.0010).

**Conclusions:**

CDO1 methylation was shown to be a strong predictor for distant metastasis in retrospective cohorts of LNP/ER+ breast cancer patients, who had received adjuvant anthracycline-based chemotherapy.

## Background

Breast cancer is the most frequent cancer in women (27% of all cancers, United States 2009), accounting for 15% of all female cancer deaths [1]. Chemotherapy of breast cancer has progressed substantially over the past decades. Anthracyclines, introduced in the 1980s, are among the most potent agents for treatment of breast cancer and thus are components of many (neo)-adjuvant and palliative regimens, more recently often in combination with taxanes [2].

In node-positive breast cancer, anthracycline-based adjuvant chemotherapy has become the standard of care since the 1990s [3]; 69% of LNP breast cancer patients remained disease-free after five years after treatment with anthracycline-based chemotherapy [4]. Those long-term disease-free patients are supposed to have been effectively treated and any more aggressive treatment thus seems to be unnecessary. Yet, treatment with anthracyclines is linked with both, acute and long-term side effects, most notably cardiotoxicity [8]. Therefore, if a biomarker was available to reliably identify LNP patients with a low risk of recurrence after adjuvant anthracycline-based chemotherapy, further treatment of this patient group with other chemotherapy agents could be avoided. Biomarkers, specifically predictive for the outcome of patients treated with anthracyclines alone, are therefore essential and will help personalize decisions regarding whether to incorporate additional chemotherapy agents into adjuvant therapy regimens for individual patients.

DNA methylation plays an important role in fundamental biological processes such as development and cellular differentiation [9]. DNA methylation has been shown to play a major role in carcinogenesis and cancer progression [10], suggesting that DNA methylation analysis may be a valuable source of predictive and/or prognostic biomarkers [11]. In this study, quantitative bisulfite sequencing [12] was used to screen 202 biomarker candidates for their prognostic impact in LNP/ER+ breast cancer patients who had received adjuvant anthracycline-based chemotherapy. The marker candidates were selected from the literature or identified by differential methylation hybridization (DMH) technology, a method for genome-wide discovery of methylation biomarkers [13]. Promoter DNA methylation of cysteine dioxygenase 1 (CDO1) was identified as a strong predictor of distant metastasis. This finding was confirmed in an independent patient group of advanced LNP/ER+ breast cancer patients treated with adjuvant anthracycline-based chemotherapy.

## Methods

### Patients

The study cohort was comprised of 162 breast cancer patients whose tumor samples were obtained from 4 clinical centers: Erasmus Medical Center, Rotterdam, The Netherlands; Centre René Huguenin, St. Cloud, France; Stiftung Tumorbank Basel, Basel, Switzerland; and Department of Obstetrics and Gynecology, Technical University of Munich, Germany. Appropriate consent, according to institutional requirements, was obtained from all patients. The study protocol was approved by the local ethics committees. Patient characteristics are shown in Table 1. All breast cancer patients were anthracycline-treated with estrogen receptor-positive, lymph node-positive tumors.

**Table 1:** Characteristics of the 162 estrogen receptor-positive and lymph node positive breast cancer patients treated with anthracyclines.

	Training Set^†^		Validation Set	
		
	All	Distant Metastasis	All	Distant Metastasis
**Total Number of Patients**	84 (100%)	39	78 (100%)	25

				
**Follow-up**				

Median follow-up [Months]	80			53.5
Range [Months]	6-144		5-166	
				
**Age at Diagnosis**				

≤ 50 Years	38 (45%)	20	41 (53%)	16
> 50 Years	46 (55%)	19	37 (47%)	19
Median Age (Years)	49		49	
Range (Years)	29-71			33 - 81
				
**T stage**				

≤ 2 cm (T1)	19 (23%)	4	24 (31%)	5
> 2 cm (T2+T3)	63 (75%)	35	53 (68%)	19
Unknown	2 (2%)	0	1 (1%)	1
				
**Tumor Grade**				

G1	2 (2%)	0	3 (4%)	1
G2	24 (29%)	11	30 (38%)	7
G3	47 (56%)	21	28 (36%)	11
Unknown	11 (13%)	7	17 (22%)	6
				
**Estrogen Receptor Status**				

Negative	0	0	0	0
Positive	84 (100%)	39	78 (100%)	25
				
**Progesterone Receptor Status**				

Negative	12 (14%)	4	18 (23%)	9
Positive	72 (86%)	35	60 (77%)	16
				
**Endocrine Treatment**				

Yes	22 (26%)	8	37 (47%)	9
No	61 (73%)	30	40 (51%)	15
Unknown	1 (1%)	1	1 (1%)	1

^†^The training set was enriched for specimens that lack PITX2 methylation.

### DNA Preparation

Leftover bisulfite DNA was used, which was prepared in the course of a previous study [14]. In brief, snap-frozen tumor tissue or tumor cell nuclei pelleted at 100,000 g were used to obtain genomic DNA as previously described [15]. Genomic DNA was extracted using the QIAamp DNA Mini Kit (Qiagen, Hilden, Germany), following the manufacturer's instructions (tissue protocol). The DNA concentration was quantified by UV spectrophotometry using a Nanodrop^® ^ND-1000 spectral photometer (Nanodrop Technologies, DE, USA). Artificially methylated DNA (CpGenome™ Universal Methylated DNA, Millipore, MA, USA) was used as completely methylated reference DNA. Two μg of extracted DNA was bisulfite converted using the EpiTect^® ^Kit (Qiagen, Hilden, Germany) according to the manufacturer's recommendations with the exception that no carrier RNA was used. DNA concentration was quantified via UV spectrophotometry as described above.

### PCR Amplification

PCR amplification was done in a 25 μl volume (1 U HotStar Taq polymerase [Qiagen, Hilden, Germany], 1 × PCR buffer [Qiagen, Hilden, Germany], 0.2 mM each dNTP [Fermentas, Burlington, Canada], 0.5 μM both primers [MWG-Biotech, Ebersberg, Germany], and 20 ng template DNA). Incubation was done using the following temperature profile: 15 min/95°C and 45 cycles with 20 s/95°C, 45 s/58°C and 30 s/72°C. The primer sequences and the sequences of the respective target loci (prior to bisulfite conversion) are listed in the Additional file 1: Analyzed genes and primer sequences. Each reverse primer contained the sequence CGTCGTCG at its 5' end.

### Sequencing and Raw Data Processing

Quantitative bisulfite sequencing was carried out as previously described [12]. ABI sequencing electropherograms were converted to text files using BioEdit 6.0.7 software and imported into Microsoft Excel. The trace containing the methylation information was visualized and the normalization signal identified. The electropherograms were shifted until the normalization signal of each sample was located at the same position. The normalization signal was integrated and each data point of the electropherogram divided by this normalization value. The analyzed PCR fragments contained several CpG sites. The signals of the single CpG sites of completely methylated DNA were used to identify the CpG positions in the electropherograms of the patient samples. The maximum intensity of a specific CpG site was defined as the maximum in the region ± 30 data points referred to the respective peak in the reference trace of the completely methylated DNA. The averaged intensities of all CpG sites from one PCR fragment were used as measurement (methylation score) for statistical analysis.

### Statistical Analysis

Time-dependent ROC curves for censored survival data and the resulting AUC were calculated according to Heagerty et al. [16]. WinSTAT for Microsoft Excel http://www.winstat.com was used for Kaplan-Meier survival analysis and log rank test. The median methylation value in the respective patient group was used as the cut point for dichotomization.

The relation between time to distant metastasis and DNA methylation score was analyzed by a linear univariate Cox Proportional Hazard model. Likelihood ratio tests were performed to test for a significant impact of DNA methylation score for the CDO1 amplificate on clinical end points. Hazard Ratios for continuous variables were calculated. Pairwise multivariate regression analysis, testing the association between clinical end point and DNA methylation score and/or clinical parameters, was performed by employing linear Cox Proportional Hazard models.

## Results

**Figure 1:**
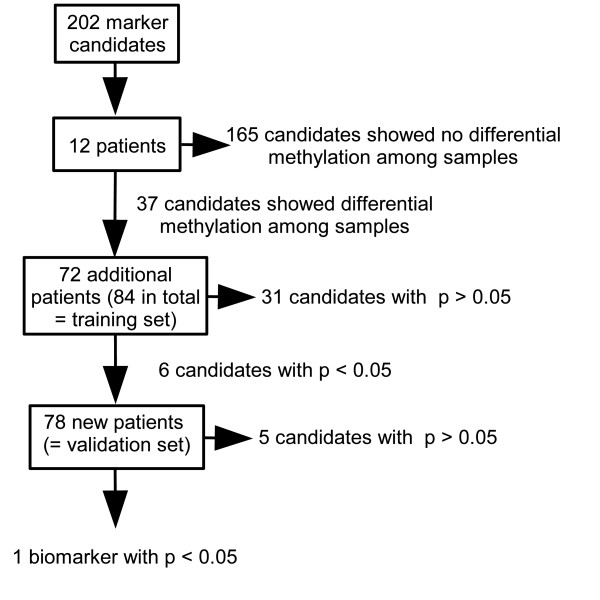
**Overview of the marker candidate selection procedure (description in text)**.

A recently published novel method for quantitative bisulfite sequencing [12] was used to analyze the methylation status of 202 potential DNA methylation biomarkers in tumors from 162 anthracycline-treated, estrogen receptor-positive, lymph node-positive breast cancer patients in order to evaluate their potential to predict distant metastasis. Information about all analyzed genes can be found in the Additional file 1: Analyzed genes and primer sequences. The marker candidates were taken from the literature or have previously been identified using differential methylation hybridization (DMH), a genome-wide discovery method (data not shown). A consecutive marker selection procedure as depicted in Figure 1 was developed in order to efficiently identify DNA methylation biomarkers for outcome prediction. In a first selection step, all 202 loci were analyzed using bisulfite treated DNA from 12 randomly selected individual tumors to exclude those that showed no evidence of differential methylation among the samples. The remaining candidates were further tested for their potential ability to predict distant metastasis in a set of 72 additional patient specimens, resulting in a training group of 84 patients in total. In the final step of analysis, the significant DNA methylation biomarkers evolving from the training set were further analyzed in an independent validation set of DNA samples from 78 patients, in order to confirm and validate their true clinical potential. The characteristics of patients belonging to the training and validation sets are shown in Table 1.

**Figure 2:**
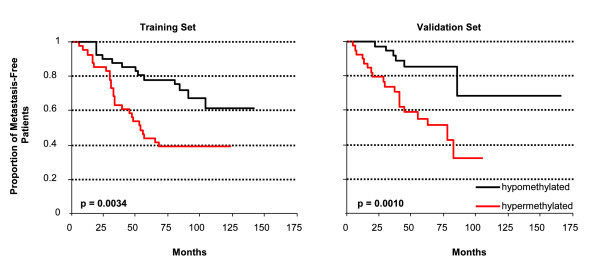
**Kaplan-Meier analysis of metastasis-free survival in the training (84 patients) and the validation set (78 patients) of lymph node-positive patients with estrogen receptor-positive tumors treated with adjuvant anthracycline containing therapy and stratified by the DNA methylation status of CDO1**. Median methylation of the respective population was used as the cut point

From the initially analyzed 202 loci, 165 did not show an apparent differential DNA methylation pattern among the initial 12 samples tested, and therefore these loci were excluded from further analyses. Of the remaining 37 candidates, six loci were associated with the occurrence of distant metastasis in this training population. The methylation data of these 37 candidates and the clinical information of the 162 patients are shown in the Additional file 2. Time-dependent ROC analysis and Kaplan-Meier analysis was performed. In order to avoid an overly optimistic result, the median DNA methylation score of the training set was used as the cut point. The results of the DNA methylation biomarkers containing prognostic information in the training set are shown in Table 2. Six genes (CDO1, APC, ZBTB16, NCR1, POU4F3, and CXCL12) emerged as potential biomarkers in the training set indicated by p < 0.05 and AUC > 0.6. Analysis of the six genes in the validation set (Table 2) confirmed the ability of the CDO1 gene to predict outcome (p = 0.0010, AUC = 0.69) while the predictive ability of DNA methylation of the other five genes could not be confirmed in the validation set, although ZBTB16 and POU4F3 just failed statistical significance. The result for CDO1 still remained significant after a Bonferroni correction for 6 tests (p = 0.0060). The Kaplan-Meier survival plots stratified by the DNA methylation status of CDO1 both in the training and validation set are depicted in Figure 2. Evidently, DNA methylation of CDO1 is a strong biomarker to predict distant metastasis in LNP patients with ER+ tumors treated with adjuvant anthracycline containing therapy. Table 3 shows the results of the univariate and the pairwise multivariate Cox Proportional Hazard models of the validation population (n = 78). In univariate analysis, the DNA methylation score for CDO1 is associated with a high risk of distant recurrence in this patient group (p = 0.0098, HR = 3.7, 95% CI 1.4 - 9.8). In addition, progesterone receptor status (p = 0.0190, HR = 2.7, 95% CI 1.2 - 6.0) was significantly associated with time-to-distant metastasis in this group whereas tumor stage, endocrine treatment, tumor grade, and age at surgery were not. CDO1 DNA methylation was a significant marker in the pairwise multivariate analysis including age at surgery, pathological T stage, progesterone receptor status, tumor grade or endocrine therapy. Patients who suffered disease recurrence showed higher DNA methylation of the CDO1 locus than those surviving metastasis-free.

**Table 2:** Time-dependent ROC analysis of the candidate genes in the training and validation set of LNP patients with ER+ tumors treated with adjuvant anthracycline containing therapy.

	Training Set (n = 84)		Validation Set (n = 78)	
		
Gene	AUC^†^	p-value^‡^	AUC^†^	p-value^‡^
CDO1	0.70	0.0034	0.69	0.0010
APC	0.68	0.0204	0.55	0.5306
ZBTB16	0.67	0.0224	0.63	0.0582
NCR1	0.63	0.0239	0.56	0.9048
POU4F3	0.69	0.0248	0.69	0.0754
CXCL12	0.67	0.0282	0.49	0.4854

^†^Shown are the AUC of the ROC at 48 months after surgery

^‡ ^The p-values are those obtained by the log rank test in Kaplan-Meier survival analysis and the genes are ranked according these p-values. The median DNA methylation score from the training set and the validation set, respectively, was used as the cut point.

**Table 3:** Univariate and pairwise multivariate Cox Proportional Hazards analysis for time-to-distant metastasis.

	Number of samples	Hazard Ratio (95% CI)	p-value^‡^
**Univariate Analysis**^†^			

CDO1 DNA Methylation	78	3.7 (1.4 - 9.8)	0.0098
Age at Surgery	78	1.3 (0.6 - 2.8)	0.5545
Tumor Stage (T2,T3 vs. T1)	78	2.0 (0.7 - 5.2)	0.1799
Progesterone Receptor Status (Positive vs. Negative)	77	2.7 (1.2 - 6.0)	0.0190
Endocrine Treatment (No vs. Yes)	77	2.0 (0.9 - 4.5)	0.1115
Tumor Grade (3 vs.1,2)	61	2.0 (0.8 - 4.9)	0.1397
**Pairwise Multivariate Analysis**^†^			

CDO1 DNA Methylation	78	3.9 (1.5 - 10.5)	0.0072
Age at Surgery	78	1.5 (0.7 - 3.4)	0.3160

			

CDO1 DNA Methylation	77	3.5 (1.3 - 9.5)	0.0128
T Stage (T2,T3 vs. T1)	77	2.0 (0.7 - 5.3)	0.1790

			

CDO1 DNA Methylation	78	3.5 (1.3 - 9.4)	0.0123
Progesterone Receptor Status (Positive vs. Negative)	78	2.5 (1.1 - 5.7)	0.0275

			

CDO1 DNA Methylation	77	4.6 (1.6 - 13.5)	0.0055
Endocrine Treatment (No vs. Yes)	77	2.0 (0.9 - 4.7)	0.0938

			

CDO1 DNA Methylation	61	3.1 (1.1 - 8.7)	0.0318
Tumor Grade (3 vs.1,2)	61	1.7 (0.7 - 4.3)	0.2506

^†^CDO1 DNA methylation and age at surgery were analyzed as continuous variables. T stage, endocrine treatment and progesterone receptor status were analyzed as binary variables.

^‡^p-values refer to Likelihood-ratio test.

A subset of the patient samples (n = 136) were also included in a previous microarray study, where DNA methylation of BMP4, FGF4, and C20orf55 was identified as biomarkers for outcome prediction [14]. The p-values obtained by the log rank test in Kaplan-Meier survival analysis revealed comparable clinical performance of the CDO1 methylation biomarker in this subgroup as compared to FGF4 (CDO1 p = 0.0017, FGF4 p = 0.0030). BMP4 and C20orf55 were not significant in this small subgroup of patients (C20orf55 p = 0.4948, BMP4 p = 0.1100). The median DNA methylation score from the 136 patient samples was used as the cut point for patient stratification.

## Discussion

DNA methylation of 202 loci was analyzed in tumors from breast cancer patients who were estrogen receptor-positive, lymph node-positive, and treated with adjuvant anthracycline-based chemotherapy, in order to identify biomarkers to predict patient outcome. Patient samples from this study were previously used to identify a four-marker panel including PITX2, BMP4, FGF4, and C20orf55 which enabled outcome prediction in lymph node-positive, HER-2-negative breast cancer patients treated with anthracycline-based chemotherapy [14].

In the presented study, cysteine dioxygenase 1 (CDO1) was identified as a strong DNA methylation biomarker for outcome prediction in the analyzed patient group. CDO1 was previously discovered as a candidate biomarker using the DMH method by determining its DNA methylation status in tumors from patients with metastatic breast cancer who were treated by FAC (5-fluorouracil, adriamycine, and cyclophosphamide) regimen as first-line therapy. The CDO1 gene encodes for an enzyme that converts cysteine to cysteine sulphinic acid and is the rate-limiting step in sulphate production. CDO1 is understood to be one of the key enzymes in the taurine biosynthetic pathway [17]. Taurine inhibits apoptosis [18-21]. The human CDO1 gene is located at chromosome 5q23.2 and is homologous to the rat and murine cysteine dioxigenases. Murine Cdo1 may be involved in the regulation of protein function and antioxidant defense mechanisms through its ability to oxidize cysteine residues [22]. Staub et al. [23] assumed that deletion or epigenetic silencing of the chromosomal region where CDO1 is located is a frequent mechanism contributing to colorectal tumorigenesis. Recently, over-expression of CDO1 was described for the Sézary syndrome, an aggressive cutaneous T-cell lymphoma [24].

Nonetheless, as of today, no aberrant DNA methylation of the CDO1 gene has been described in the context of breast cancer. Expression of cysteine dioxygenase was found in ductal cells of pregnant rats, but not in other mammary epithelial cells or in ductal cells of nonpregnant rats [25]. Interestingly, repression of Cdo1 expression was identified to be associated with the malignant transition from mammary intraepithelial neoplasia to tumors in an engineered mouse-based model of ductal carcinoma in situ [26]. However, whether the observed repression was caused by DNA methylation of CDO1 was not assessed in that study.

The results of this study do not show if methylation of CDO1 is a general prognostic biomarker which is independent of the nature of the adjuvant treatment or if it is predictive for a response to an adjuvant anthracycline-based chemotherapy. Further studies with other patient populations such as patients who did not receive an adjuvant anthracycline-based chemotherapy or the functional analysis of cell lines might shed further light on a potential predictive value of CDO1 methylation.

## Conclusions

DNA methylation of CDO1 was found to be a strong biomarker for prediction of distant recurrence in lymph node-positive patients with estrogen receptor-positive tumors treated with adjuvant anthracycline containing therapy.

## Abbreviations

ROC: Receiver Operating Characteristic; AUC: Area under the Curve; DMH: Differential Methylation Hybridization; CI: Confidence Interval; HR: Hazard Ratio; CDO1: Cysteine Dioxygenase 1; APC: Adenomatosis Polyposis Coli; MDA: Multiple Displacement Amplification; NCR1: Natural Cytotoxicity Triggering Receptor 1; POU4F3: POU Class 4 Homeobox 3; CXCL12: Chemokine; (C-X-C motif) Ligand 12; ZBTB16: Zinc Finger and BTB Domain Containing 16.

## Competing interests

The authors DD, MK, AF, JD, JL, and RL are or have been employees and/or shareholders of Epigenomics AG (Berlin, Germany), a company that aims to commercialize DNA methylation markers. The other authors report to have no conflict of interest regarding the topic of the article.

## Authors' contributions

DD drafted the manuscript, conceived and coordinated the study, participated in marker candidate selection, helped to carry out the experiments and participated in the statistical analysis. MK carried out the statistical analysis, participated in carrying out the experiments and helped to draft the manuscript. JD developed scripts for the data management, data processing and analysis and helped to carry out the experiments and to draft the manuscript. JL provided a script for the conversion of sequencing raw data into txt format. AF provided marker candidates from DMH studies. NH, MS, SEC, VV, FS, and JAF participated in the design of the study and provided and characterized the sample material, and assisted in the preparation of the manuscript. JWMM participated in the design of the study, provided and characterized the sample material and finalized the manuscript. RL participated in the design of the study and its supervision, provided marker candidates and revised the manuscript. All authors read and approved the final version of the manuscript.

## Pre-publication history

The pre-publication history for this paper can be accessed here:

http://www.biomedcentral.com/1471-2407/10/247/prepub

## Supplementary Material

Additional file 1 **Analyzed genes and primer sequences**. This excel spreadsheet (.xls) contains the names of the analyzed genes, the primer sequences and the sequences of the analyzed regions. Click here for file

Additional file 2 **DNA methylation data and patient information**. This excel spreadsheet (.xls) contains clinical patient information from 162 patients and the DNA methylation data from 37 genes which showed differential methylation among the first analyzed 12 samples. Click here for file
